# A Systematic Review on the Antimicrobial Activity of Andrographolide

**DOI:** 10.4014/jmb.2408.08028

**Published:** 2024-11-15

**Authors:** Gayus Sale Dafur, Aiza Harun, Tuan Noorkorina Tuan Kub, Ruzilawati Abu Bakar, Azian Harun

**Affiliations:** 1 Department of Medical Microbiology and Parasitology, School of Medical Sciences, Universiti Sains Malaysia, Kubang Kerian, Kelantan 16150, Malaysia; 2 Department of Biology, Federal College of Education Pankshin, Plateau State 933105, Nigeria; 3 Faculty of Applied Sciences, Universiti Teknologi MARA, Bandar Jengka, Bandar Tun Razak 26400, Pahang; 4 Department of Pharmacology, School of Medical Sciences, Universiti Sains Malaysia, Kubang Kerian, Kelantan 16150, Malaysia; 5 Hospital Universiti Sains Malaysia, Jalan Raja Peremppuan Zainab II, Kubang Kerian, Kelantan 16150, Malaysia

**Keywords:** Antimicrobial activity, andrographolide, systematic review

## Abstract

Andrographolide, a bioactive compound from *Andrographis paniculata*, has gained attention for its antimicrobial properties, which include antibacterial, antiviral, antifungal, and antiprotozoal effects. As an herbal extract used in traditional medicines, andrographolide also shows promise for developing new antimicrobial agents, especially in the fight against rising antimicrobial resistance. Following the PRISMA 2020 guidelines, 16 peer-reviewed studies published from 2010 to 2024 and focusing on andrographolide’s effects on bacteria, viruses, fungi, and protozoa were reviewed. The quality and bias risk of these studies were assessed using the In Vitro Quality Evaluation Instrument to ensure methodological rigor. The findings demonstrate that andrographolide is effective against bacteria such as *Escherichia coli*, *Klebsiella pneumoniae*, and *Staphylococcus aureus*. However, its antifungal efficacy is limited, as it was ineffective against *Candida albicans* and *Saccharomyces cerevisiae*, but effective against *Alternaria solani*. It exhibited strong antiviral activity against 2019-nCoV, Dengue virus, and Enterovirus D68, and showed antiprotozoal effects against *Plasmodium falciparum* and *Setaria cervi*. Nonetheless, variations in its efficacy across different microorganisms were observed. The quality assessment revealed low bias risk in 11 out of 16 studies (78.57% to 92.86%), while the remaining five had medium bias risk (57.14% to 64.29%), indicating an overall acceptable quality of the studies. Information on andrographolide’s potential and effectiveness across various microorganisms is crucial. Therefore, the purpose of this review was to synthesize the existing data on andrographolide’s antimicrobial activity and assess its potential in combating antimicrobial resistance. This review highlights the need for further research on andrographolide’s antifungal activity, mechanisms of action, clinical safety, toxicity, and potential applications in antimicrobial resistance strategies.

## Introduction

Medicinal plants are of vast importance to human health [1]. It is well known that herbal extracts can be used to treat a variety of infectious and non-infectious disorders [1, 2]. Novel antimicrobials can be found in plants [3], and those used in traditional medicines include natural and active antimicrobial compounds that are often affordable, safe, and efficient for treating common diseases with microbial origins [4, 5]. According to reports, medicinal plants are the most abundant source of modern medications, traditional medicines, folk medicines, ingredients for synthetic drugs, and pharmaceutical intermediates [6]. New compounds with antimicrobial characteristics are constantly being discovered in plants [7, 8]. Additionally, the discovery and characterization of active components in medicinal plants has tremendously aided in developing innovative medications with potent therapeutic effects to address a range of medical conditions [9]. Numerous plants contain various active ingredients with wide-ranging therapeutic and antimicrobial characteristics. It is therefore crucial to explore these plants and their constituents to develop new antimicrobial drugs, as well as to discover and publicize their therapeutic efficacy.

Andrographolide is a labdane diterpenoid bioactive compound primarily derived from the *Andrographis paniculata* plant. Due to its diverse biological activities, this plant is extensively utilized in conventional medicine to treat numerous illnesses or infections [10-14]. Andrographolide is shown in numerous studies to have efficacy against a variety of microbiological infections. It exerts antiviral [15-22], antibacterial [23-26], antifungal [27], and antiprotozoal activities [28, 29], making it a viable option for creating new antimicrobial drugs. However, there is little available information and no thorough analysis has been conducted on andrographolide's effectiveness as an antimicrobial agent, including its mode of action or molecular interactions with microorganisms.

In this review, we aimed to assess, synthesize, and summarize the available evidence on the antimicrobial activity of andrographolide against diverse microbes, such as viruses, bacteria, fungi, and protozoa to uncover the current evidence on this topic. Previously, the antimicrobial activity of andrographolide was the subject of a review that evaluated the methodological merits and conclusions of many original research publications and gave an overview of how each of these classes of microorganisms was affected by andrographolide. This review is important because andrographolide has shown promise traditionally in treating various illnesses, as well as in studies performed in vitro against some microorganisms. Unfortunately, the lack of related comprehensive data has prevented determining the spectrum of antimicrobial activity that the compound possesses against microbiological pathogens. In addition, our review offers important information about the effectiveness, spectrum of action, and possible uses of andrographolide against different microbial pathogens, which could help solve a major and pressing problem in public health and microbiology. This information might result in the creation of novel antimicrobial therapies, especially considering the growing concerns about antimicrobial resistance and global health. Finally, this review identifies knowledge gaps that should be filled to direct future studies and enhance both public health and microbiological research.

## Characteristics and Advantages of Andrographolide

Andrographolide, a key bioactive compound sourced from *Andrographis paniculata* plants, serves as a natural alternative to synthetic antimicrobial agents. It offers the advantage of potentially fewer side effects and a reduced risk of promoting resistance. Its long history in traditional medicine underscores its safety. Andrographolide is a colorless, crystalline substance with a distinctly bitter taste and is noted for its diverse biological activities. Its traditional medicinal uses and promising antimicrobial properties, which include antiviral, antibacterial, antifungal, and antiprotozoal effects, make it a strong contender for developing new antimicrobial agents, particularly in combating the growing threat of antimicrobial resistance.

## Review Approach and Scope

### Design of the Study

**Fig. 1:**
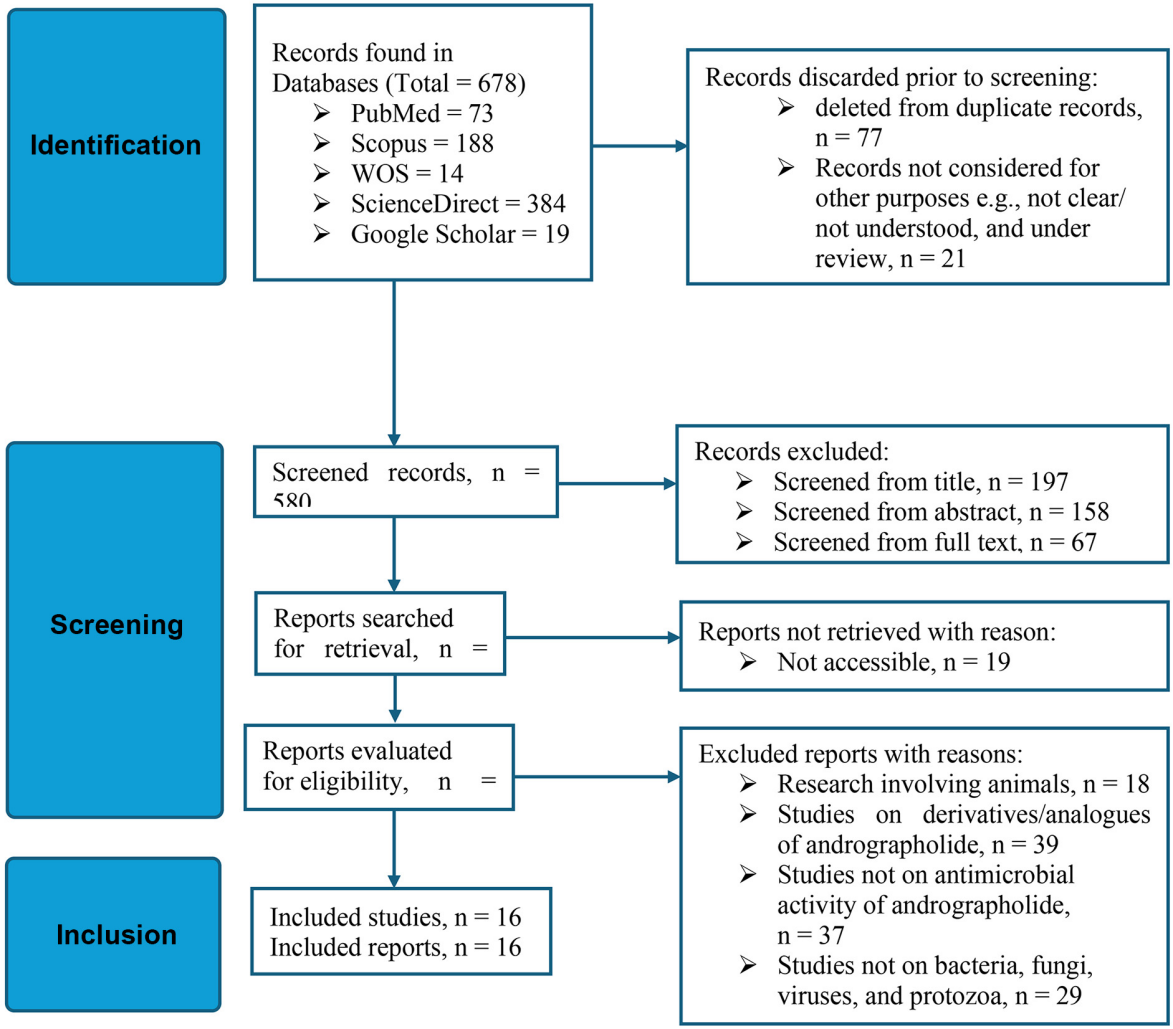
PRISMA flow diagram of articles screened for inclusion in the systematic review.

The Preferred Reporting Items for Systematic Reviews and Meta-Analyses (PRISMA) 2020 guideline [30] was adopted for this systematic review (Fig. 1). PRISMA 2020 is a framework designed to enhance transparency and completeness in systematic reviews by providing a structured checklist and flow diagram for reporting. In this review, PRISMA ensures the clear documentation of study selection, search strategy, risk of bias assessment, and data synthesis, making the review reproducible and comprehensive. Unlike other types of reviews, PRISMA emphasizes standardized reporting, detailed risk of bias assessment, and thorough exploration of heterogeneity among studies. Its application in this study ensures that the review process meets the highest standards of scientific rigor, promoting transparency, replicability, and credibility in the findings.

## Inclusion Criteria

The review included studies that fulfilled the following criteria:

i. In vitro and ex vivo studies evaluating the antimicrobial activity or effect of andrographolide or diterpene lactone against viruses, bacteria, fungi, and protozoa.

ii. Publications with peer review that were published in the English language between January 2010 and January 2024.

iii. Original study findings published in scientific journals.

## Exclusion Criteria

The following criteria were used to eliminate studies:

i. Studies that use animals.

ii. Studies not evaluating the antimicrobial activity or effect of andrographolide or diterpene lactone.

iii. Studies not involving bacteria, fungi, viruses, or protozoa.

iv. Studies that used derivatives or analogues of andrographolide.

## Search Strategy

Using the terms "antimicrobial" OR "antifungal" OR "antibacterial" OR "antiviral" OR "antiprotozoal" AND "activity" OR "effect" AND "andrographolide" OR "diterpene lactone," we conducted an extensive electronic database search across PubMed, Web of Science, Scopus, Google Scholar, and ScienceDirect to look for articles or studies from January 2010 to January 2024. The search was limited to original, peer-reviewed articles that were written in English. Additionally, we manually reviewed the collected publications' reference lists for any additional pertinent studies.

## Search Outcome

In all, 678 papers that discussed andrographolide's antimicrobial properties. A total of 580 peer-reviewed publications were screened after the selection criteria were applied. Then, after duplicates, articles under review, and articles discarded for other reasons were removed, 158 articles were sought for retrieval. Among these, 139 articles were evaluated for eligibility, and 19 articles could not be downloaded since their full texts were not available (Fig. 1). Out of the 139 publications evaluated, 16 peer-reviewed articles met the criteria for inclusion, and thus were included in this review. The title, abstract, and full text of each peer-reviewed paper were independently assessed by all authors to determine the research eligibility. Through group discussion, any discrepancies or conflicts in the authors' eligibility assessment were settled.

## Quality Assessment of Included Studies

The quality and bias risk of each included study were assessed using the 12 items or criteria of the In Vitro Quality Evaluation Instrument (QUIN TOOL) of [31], as shown in Table 1. Three reviewers independently evaluated each study, while two reviewers provided confirmation for each assessment. The reviewers discussed any differences or disagreements, and reached a consensus as a group. Each study was evaluated by assigning one of the following scores to every one of the twelve items: 2 for adequately stated, 1 for inadequately stated, 0 for unspecified, and criteria were removed from computation for unapplicable items. To determine the total rating for a specific study, the scores were added together and used to determine the final percentage (%) score, according to the formula: Final percentage (%) score = (overall score / 2 × number of relevant criteria) × 100. Final percentage (%) scores obtained were utilized to categorize each of the studies as low risk of bias (70% and above), medium risk of bias (between 50% and 70%), or high risk of bias (below 50 %).

The assessment of bias risk determines the degree to which a study's design and methodology are free from any potential bias that could have an impact on the study's findings. Thus, determining the genuine impact of the test carried out from a specific application requires evaluating the risk of bias in a study [32].

## Data Extraction and Synthesis

The eligibility of the publications was evaluated independently by three reviewers by looking at their titles, abstracts, and complete texts. Any differences or disagreements were discussed or settled with the assistance of the other two reviewers. A typical data extraction form (Table 2) with the following details was used to extract the research findings that were included:

i. Author(s) and publication year.

ii. Type of research (in vitro or ex vivo).

iii. Type of microorganism (bacteria, fungi, viruses, or protozoa).

iv. Effective concentration.

v. Method of evaluation and results (*i.e.*, the antimicrobial activity or effect).

## Overview of the Included Studies

This comprehensive review included 16 studies overall (15 studies conducted in vitro and one ex vivo study). The studies evaluated andrographolide's antimicrobial action against a range of microorganisms, such as viruses, bacteria, fungi, and protozoa (Table 2). Of these studies, two assessed andrographolide's antimicrobial efficacy against bacteria [23, 26], two examined its antimicrobial activity against both bacteria and fungi [24, 25], one study evaluated its antimicrobial activity against only a fungus [27], eight studies investigated its antimicrobial activity against viruses [15-22], and three studies evaluated its antimicrobial activity against protozoa [28, 29, 33].

## Efficacy of Andrographolide against Different Microorganisms

This review evaluated the antimicrobial activity of andrographolide against a broad variety of microbes, such as viruses, protozoa, bacteria, and fungi. Most of the studies (Table 2) reported that andrographolide exhibited significant antimicrobial activity against various strains of bacterial species, including *Staphylococcus aureus*, *Streptococcus thermophilus*, *Bacillus subtilis*, *Mycobacterium smegmatis*, *Pseudomonas aeruginosa*, *Escherichia coli*, *Klebsiella pneumoniae*, *Neisseria gonorrhoeae*, *Streptococcus pyogenes*, *Salmonella paraty-phi* B, and *Vibrio cholerae* [23-26].

However, *Proteus mirabilis*, *Enterococcus faecalis*, and *Staphylococcus aureus* were found to be resistant to it even at high concentration as established in the study conducted by [25]. Andrographolide was also found to be effective against the fungus *Alternaria solani* [27], but was found ineffective against *Saccharomyces cerevisiae* and *Candida albicans* [24, 25]. In addition, andrographolide showed antiviral activity against several viruses, including Human Coronavirus, Dengue virus, Enterovirus D68, Foot-and-Mouth-Disease virus, Hepatitis C virus, Severe Acute Respiratory Syndrome Coronavirus (SARS-CoV), 2019 Novel Coronavirus (2019-nCoV), and Chikungunya virus [15-22]. Furthermore, andrographolide exhibited antiprotozoal activity against certain protozoa, including *Plasmodium falciparum* and *Setaria cervi* [28, 29], but was found to be ineffective against *Theileria equi* [33]. Depending on the microbial strain and the experimental settings, andrographolide's antimicrobial effectiveness varies against several types of microbes.

## Quality of the Included Studies

The quality of each study was assessed using the Quality Evaluation Instrument for In Vitro Studies [31]. This tool was utilized to evaluate the bias risk of individual studies included in the current review. Out of the sixteen (16) studies that make up this review (Table 3), 11 (68.75%) were evaluated to have less bias risk with the percentage score range of 78.57%-92.86% [15-22, 24, 28, 29], whereas 5 (31.25%) studies had medium risk of bias with the percentage score of between 57.14% and 64.29% [23, 25-27, 33]. There was no significant bias risk associated with any of the examined studies. This suggests that the overall caliber of the research covered in this review was acceptable.

## Summary and Overall Outcome of the Review

Andrographolide has been shown in numerous studies to have antibacterial action against a variety of harmful bacteria. One study [24] revealed that at minimum inhibitory concentration (MIC) ranges of 100-350 μg/ml, andrographolide demonstrated a broad range of growth inhibition activity against *Bacillus subtilis*, *Escherichia coli*, *Mycobacterium smegmatis*, *Klebsiella pneumoniae*, *Pseudomonas aeruginosa*, *Staphylococcus aureus*, and *Streptococcus thermophilus*. Another study [26] uncovered the antibacterial activity of andrographolide against *Klebsiella pneumoniae*, *Escherichia coli*, *Pseudomonas aeruginosa*, *Staphylococcus aureus*, and *Streptococcus pyogenes* at MIC values ranging between 125 μg/ml and 250 μg/ml. These studies also showed that *Neisseria gonorrhoeae* was significantly susceptible to andrographolide at an MIC value of 60 μg/ml. In addition, a study by [23] confirmed that at a dosage of 3.0 mg/ml, andrographolide displayed antibacterial activity against *Escherichia coli*. Conversely, the study of [25] established that *Proteus mirabilis*, *Enterococcus faecalis*, and *Staphylococcus aureus* were resistant to andrographolide, even at the highest concentration of 875 μg/ml. However, they also noted in the same investigation that andrographolide demonstrated bactericidal and inhibitory effects on *Salmonella enterica* ser. Paratyphi B, *Streptococcus pyogenes*, *Klebsiella pneumoniae*, and *Vibrio cholerae*.

According to the review's findings, there is a dearth of research on andrographolide's antifungal activity since the compound's effectiveness against fungi has only been looked at in a limited number of studies. However, [24] reported in their investigation that andrographolide had no effect on the yeasts *Candida albicans* and *Saccharomyces cerevisiae*. This was supported by [25], who also established that *C. albicans* was resistant to andrographolide, even at the concentration of 875 μg/ml. In a contrary report, andrographolide was found to exhibit inhibitory activity against *Alternaria solani* spore germination by 64.8% at a concentration of 500 mg/l [27]. This implies that more research is needed on the antifungal activity of andrographolide.

Andrographolide was found to exhibit antiviral activity against several viruses. It suppressed the protease activities of 2019-nCoV M^pro^ and SARS-CoV M^pro^ at the half-maximal inhibitory concentration (IC_50_) values of 15.05 ± 1.58 μM, and 5.00 ± 0.67 μM, respectively [20]. This was corroborated by the findings of [16], who also reported the anti-Human coronavirus (anti-HCoV-OC43) activity of andrographolide at concentrations between 0.92 mg/ml and 2.34 mg/ml. Moreover, a study by [19] recorded that andrographolide demonstrated anti-dengue virus (anti-DENV) activity at the half-maximal effective concentration (EC_50_) values of 21.304 μM and 22.739 μM. This was also recorded by [18], who found a significant reduction in dengue virus (DENV) titer in response to andrographolide treatment at 50 μM concentration. In addition, andrographolide was found to greatly reduce Enterovirus D68 (EV-D68) RNA replication and had antiviral efficacy against EV-D68 infection at the EC_50_ value of 3.45 μM [15]. The research of [21] reported that andrographolide demonstrated inhibitory action against the virus causing foot-and-mouth disease (FMDV) at the median effective dose value (ED_50_) of 52.18 ± 0.01 μM. Also reported by [17], andrographolide considerably decreased hepatitis C virus (HCV) RNA levels at an EC_50_ value of 6.0 ± 0.5 μM. Furthermore, a study by [22] established that the compound demonstrated good suppression of infection with chikungunya virus (CHIKV) and decreased the virus production at an EC_50_ value of 77 μM.

In this review, andrographolide has also been associated with antiprotozoal activity against a few protozoan parasites. As reported by [29], andrographolide was found to significantly (*p* < 0.05) decrease the proportion of red blood cells (RBCs) with *Plasmodium* ring infection despite its less pronounced anti-plasmodium action. Also, in corroboration of the antiprotozoal activity of andrographolide, [28] established that the compound demonstrated potential anti-filarial activity against *Setaria cervi* (a bovine filarial parasite) at an IC_50_ value of 24.80 μM. On the other hand, andrographolide was found to be ineffective against *Theileria equi* parasites, even at the highest concentration of 100 μM [33].

According to the findings of this thorough review, andrographolide may be useful as a natural antimicrobial agent against various microorganisms, despite some differences recorded in the results of the included studies. These differences could be due to a variety of factors, such as strain variances, compound potency, methodological approach, and other variables.

## Conclusion

Andrographolide appears to have strong antimicrobial activity against a variety of microbial strains, as we have reported in this review according to the available information. This compound is a viable candidate for the development of new therapeutic drugs due to its ability to reduce and hinder the growth and activity of bacterial pathogens, some fungal and protozoal pathogens, and the replication and proliferation of viral pathogens. However, additional study is required to assess and clarify andrographolide’s efficacy against a wide variety of fungal pathogens, such as *Aspergillus* species, *Trichophyton* species, *Microsporum* species, among others, as only a few studies were found on the antifungal activity of the compound, and these were limited to only *C. albicans*, *S. cerevisiae*, and *Alternaria solani*. Additionally, more studies are needed to investigate the mechanisms of action, toxicity, and safety of andrographolide in clinical trials, as well as its potential side effects and drug interactions.

## Figures and Tables

**Table 1:** The score distribution and evaluation parameters for bias risk in the included studies.

S/N	Criteria/Check list			
	Adequately stated (Score = 2)	Inadequately stated (Score = 1)	Unspecified (Score = 0)	Unapplicable (Excluded)
1	Clearly defined aims or objectives			
2	A thorough description of the sample size calculation			
3	Detailed description of sampling method			
4	Information about the comparison group			
5	Detailed description of the methodology			
6	Operator details			
7	Randomization			
8	Measurement of results method			
9	Details of the outcome assessor			
10	Blinding			
11	Statistical analysis			
12	Results presentation			

**Table 2:** Information extracted from the included studies.

Authors (year)	Research type	Type(s) of microorganisms	Effective concentration	Evaluation method	Results
Zaid *et al*. (2015) [29]	In vitro	Protozoan: *Plasmodium falciparum*	Drug sensitivity assay: IC_50_ =1039.3 ± 45.76 nM, IC_90_ = 2102.4 ± 56.2 nM Merozoite invasion assay: > 125 nM	Conventional malaria drug sensitivity assay and merozoite invasion assay	Andrographolide's anti-plasmodi um action was less pronounced in the drug sensitivity assay, although it significantly (*p* < 0.05) decreased the proportion of RBCs with plasmodium ring infection in the merozoite invasion experiment.
Komaikul *et al*. (2023) [16]	In vitro	Virus: Human coronavirus (HCoV-OC43)	Ranges between 0.92 mg/ml - 2.34 mg/ml	Cell viability and in-cell ELISA tests	The anti-HCoV-OC43 action of the 2.34 mg/ml andrographolidecontaining methanolic extract was less compared to DES extracts with less andrographolide contents of 0.92 to 1.46 mg/ml.
Yadav *et al*. (2022) [28]	Ex vivo	Protozoan: Bovine filarial parasite (*Setaria cervi*)	IC_50_ value of 24.80 μM	MTT [3-(4,5-Dimethylthiazol-2-yl)-2,5-Diphenyltetrazolium Bromide] assay	With an IC_50_ value of 24.80 μM determined by the MTT experiment, andrographolide demonstrated potential antifilarial activity.
Paemanee *et al*. (2019) [18]	In vitro	Virus: Dengue virus (DENV)	50 μM	Cells lines culture and standard plaque assay	A significant reduction in the virus titer in response to andrographolide treatment was revealed with 50 μM concentration.
Arifullah *et al*. (2013) [24]	In vitro	Bacteria/Fungi: *Escherichia coli*, *Bacillus subtilis*, *Mycobacterium smegmatis*, *Staphylococcus aureus*, *Streptococcus thermophilus* *Pseudomonas aeruginosa*, and *Klebsiella pneumoniae* Fungi (Yeasts): *Candida albicans*, *Saccharomyces cerevisiae*	50 μg/ml- 350 μg/ml	Modified agar-disc diffusion method and broth microdilution method	Andrographolide demonstrated a broad range of growth inhibition activity with MIC values range between 50 μg/ml to 350 μg/ml against the bacteria *Bacillus subtilis*, *Staphylococcus aureus*, *Streptococcus thermophilus*, *Escherichia coli*, *Mycobacterium smegmatis*, *Klebsiella pneumoniae*, and *Pseudomonas aeruginosa*. When tested on the yeasts *Candida albicans* and *Saccharomyces cerevisiae*, it proved ineffective.
Panraksa *et al*. (2017) [19]	In vitro	Virus: Dengue virus (DENV)	21.304 μM and 22.739 μM	Cells lines culture and standard plaque assay.	Both the 21.304 μM and 22.739 μM 50% effective doses (EC_50_) of andrographolide demonstrated anti-DENV activity.
Bassey *et al*. (2021) [26]	In vitro	Bacteria: *Pseudomonas aeruginosa*, *Klebsiella pneumoniae*, *Escherichia coli*, *Streptococcus pyogenes*, and *Staphylococcus aureus*.	60 μg/ml - 250 μg/ml	Broth microdilution method	*Neisseria gonorrhoeae* was shown to be markedly susceptible to andrographolide (CF6) with 60 μg/ ml (MIC value), whereas *S. pyogenes* and *S. aureus* displayed 125 μg/ml MIC value each. At a concentration of 250 μg/ml, bacteria that are gram-negative, including *E. coli*, *K. pneumonia*, and *P. aeruginosa* were all suppressed.
Wang *et al*. (2018) [15]	In vitro	Virus: Enterovirus D68 (EV-D68)	EC_50_ = 3.45 μM	Cell culture, cell viability assay, and endpoint dilution assay (EPDA).	With an EC_50_ of 3.45 μM, andrographolide greatly reduced EV-D68 RNA replication and had antiviral efficacy against EV-D68 infection.
Nidiry *et al*. (2015) [27]	In vitro	Fungi: *Alternaria solani*	500 mg/l	Spore germination inhibition study.	At a 500 mg/l concentration, andrographolide inhibits *Alternaria solani* spore germination by 64.8%.
Theerawata nasirikul *et al*. (2022) [21]	In vitro	Virus: Foot-and-mouth-disease virus (FMDV)	52.18 ± 0.01 μM	Cell culture, antiviral activity assays, and RTqPCR.	Effective concentration (EC_50_) value of 52.18 ± 0.01 μM was observed for andrographolide's inhibitory activity as measured by RT-qPCR.
Lee *et al*. (2014) [17]	In vitro	Virus: Hepatitis C virus (HCV)	6.0 ± 0.5 μM	Cell culture, western blotting, and qRT-PCR analysis.	With an effective concentration (EC_50_) value of 6.0 ± 0.5 μM, andrographolide considerably decreased HCV RNA levels.
Ali and Ahmad Mir (2020) [23]	In vitro	Bacteria: *E. coli* MTCC1679	3.0 mg/ml	Agar well diffusion method for antibacterial testing.	The bacterial pathogen (*Escherichia coli* MTCC1679) was inhibited by the andrographolide compound with a 12 ± 1.0 mm zone of inhibition at a concentration of 3.0 mg/ml.
Gopalakris hnan *et al*. (2016) [33]	In vitro	Protozoan: *Theileria equi*	Highest concentrati on was 100 μM	In vitro growth inhibition assay.	Andrographolide was ineffective in inhibiting the growth of *T. equi* parasites even at the highest concentration of 100 μM used.
Shi *et al*. (2020) [20]	In vitro	Viruses: 2019 novel corona virus (2019-nCoV) and severe acute respiratory syndrome coronavirus (SARS-CoV)	2019-nCoV: 15.05 ± 1.58 μM SARS-CoV: 5.00 ± 0.67 μM	Protease activity assay.	The 2019-nCoV M^pro^ protease activity was inhibited by andrographolide at an IC_50_ of 15.05 ± 1.58 μM, while SARS-CoV M^pro^ was suppressed at an IC_50_ of 5.00 ± 0.67 μM.
Ativui *et al*. (2022) [25]	In vitro	Bacteria: *Salmonella* Paratyphi B, *Enterococcus faecalis*, *S. pyogenes*, *E. coli*, *Vibrio cholerae*, *Pseudomonas aeruginosa*, *Staphylococcus aureus*, *Proteus mirabilis*, *Klebsiella pneumoniae* Fungus (Yeast): *C. albicans*	1.71 μg/ml and 875 μg/ml	Highthroughput spot culture growth inhibition (HTSPOTi) technique (anti-infective assay).	While resistance was observed in *Staphylococcus aureus*, *Proteus mirabilis*, *Enterococcus faecalis*, and *Candida albicans* even at 875 μg/ml concentration, andrographolide exhibited bactericidal activity against *Klebsiella pneumoniae*, *Streptococcus pyogenes*, *Salmonella paratyphi* B, with the maximum inhibitory activity of 1.71 μg/ml against *Vibrio cholerae*.
Wintachai *et al*. (2015) [22]	In vitro	Virus: chikungunya virus (CHIKV)	EC_50_ value of 77 μM	Cell lines culture, virucidal and standard plaque assays.	With an effective concentration (EC_50_) of 77 μM, andrographolide showed good suppression of CHIKV infection and decreased virus production by about 3 log_10_.

**Table 3:** Quality assessment and risk of bias detection for the included studies.

Authors	Criteria/check lists														
	1	2	3	4	5	6	7	8	9	10	11	12	Overall score	Score (%)	Risk of bias
Zaid *et al*. (2015) [29]	2	-	-	1	2	-	-	2	-	1	1	2	11	78.57	Low
Komaikul *et al*. (2023) [16]	2	-	-	1	2	-	-	2	-	1	2	2	12	85.71	Low
Yadav *et al*. (2022) [28]	2	-	-	1	2	-	-	2	-	1	2	2	12	85.71	Low
Paemanee *et al*. (2019) [18]	2	-	-	2	2	-	-	2	-	1	2	2	13	92.86	Low
Arifullah *et al*. (2013) [24]	2	-	-	2	2	-	-	2	-	1	2	2	13	92.86	Low
Panraksa *et al*. (2017) [19]	2	-	-	2	2	-	-	2	-	0	2	2	12	85.71	Low
Bassey *et al*. (2021) [26]	0	-	-	1	2	-	-	2	-	1	1	2	9	64.29	Medium
Wang *et al*. (2018) [15]	1	-	-	1	2	-	-	2	-	1	2	2	11	78.57	Low
Nidiry *et al*. (2015) [27]	1	-	-	1	2	-	-	2	-	0	1	2	9	64.29	Medium
Theerawatanasirikul *et al*. (2022) [21]	1	-	-	2	2	-	-	2	-	1	1	2	11	78.57	Low
Lee *et al*. (2014) [17]	1	-	-	2	2	-	-	2	-	1	2	2	12	85.71	Low
Ali and Ahmad Mir (2020) [23]	0	-	-	2	2	-	-	2	-	0	0	2	8	57.14	Medium
Gopalakrishnan *et al*. (2016) [33]	1	-	-	2	2	-	-	2	-	0	0	2	9	64.29	Medium
Shi *et al*. (2020) [20]	2	-	-	0	2	-	-	2	-	1	2	2	11	78.57	Low
Ativui *et al*. (2022) [25]	2	-	-	0	2	-	-	2	-	0	0	2	8	57.14	Medium
Wintachai *et al*. (2015) [22]	0	-	-	2	2	-	-	2	-	1	2	2	11	78.57	Low

Unapplicable items not included in the computation (-).

## References

[1] Murtaza G, Mukhtar M, Sarfraz A (2015). A review: antifungal potentials of medicinal plants. J. Bioresour. Manage..

[2] Górniak I, Bartoszewski R, Króliczewski J (2019). Comprehensive review of antimicrobial activities of plant flavonoids. Phytochem. Rev..

[3] Webster D, Taschereau P, Belland RJ, Sand C, Rennie RP (2008). Antifungal activity of medicinal plant extracts; preliminary screening studies. J. Ethnopharmacol..

[4] Begum HA, Asad F, Hamayun M, Murad W, Khan A, Yaseen T (2021). Antifungal activity of six medicinal plants of Pakistan against selected fungi. Bangladesh J. Bot..

[5] Mathur R (2012). Antimicrobial potential and phytochemical analysis of plant extracts of *Anethum sowa*. Int. J. Curr. Res. Rev..

[6] Hailu T, Bachheti RK, Dekebo A (2016). Phytochemical analysis and antimicrobial activity of *Senna didymobotrya* seed extracts. Der. Pharma Chem..

[7] Aboh MI, Olayinka BO, Adeshina GO, Oladosu P (2014). Antifungal activities of Phyto compounds from *Mitracarpus villosus* (sw.) Dc from Abuja, Nigeria. J. Microbiol. Res..

[8] Aboh MI, Oladosu P, Adeshina GO, Olayinka BO, Olonitola S, Atasie NH (2018). Screening of selected medicinal plants for their antifungal properties. Afr. J. Clin. Exp. Microbiol..

[9] Hassan A, Ullah H, Bonomo MG (2019). Antibacterial and antifungal activities of the medicinal plant *Veronica biloba*. J. Chem..

[10] Chan SJ, Wong WSF, Wong PTH, Bian JS (2010). Neuroprotective effects of andrographolide in a rat model of permanent cerebral ischaemia. Br. J. Pharmacol..

[11] Dai Y, Chen SR, Chai L, Zhao J, Wang YY, Wang YY (2019). Overview of pharmacological activities of *Andrographis paniculata* and its major compound andrographolide. Crit. Rev. Food Sci. Nutr..

[12] Jayakumar T, Hsieh CY, Lee JJ, Sheu JR (2013). Experimental and clinical pharmacology of *Andrographis paniculata* and its major bioactive phytoconstituent andrographolide. Evid. Based Complement. Alternat. Med..

[13] Lin HC, Lii CK, Chen HC, Lin AH, Yang YC, Chen HW (2018). Andrographolide inhibits oxidized LDL-induced cholesterol accumulation and foam cell formation in macrophages. Am. J. Chin. Med..

[14] Maiti K, Mukherjee K, Murugan V, Saha BP, Mukherjee PK (2010). Enhancing bioavailability and hepatoprotective activity of andrographolide from *Andrographis paniculata*, a well-known medicinal food, through its herbosome. J. Sci. Food Agric..

[15] Wang D, Guo H, Chang J, Wang D, Liu B, Gao P (2018). Andrographolide prevents EV-D68 replication by inhibiting the acidification of virus-containing endocytic vesicles. Front. Microbiol..

[16] Komaikul J, Ruangdachsuwan S, Wanlayaporn D, Palabodeewat S, Punyahathaikul S, Churod T (2023). Effect of andrographolide and deep eutectic solvent extracts of *Andrographis paniculata* on human coronavirus organ culture 43(HCoV-OC43). Phytomedicine.

[17] Lee JC, Tseng CK, Young KC, Sun HY, Wang SW, Chen WC (2014). Andrographolide exerts anti-hepatitis C virus activity by upregulating haeme oxygenase-1 via the p38 MAPK/Nrf2 pathway in human hepatoma cells. Br. J. Pharmacol..

[18] Paemanee A, Hitakarun A, Wintachai P, Roytrakul S, Smith DR (2019). A proteomic analysis of the anti-dengue virus activity of andrographolide. Biomed. Pharmacother..

[19] Panraksa P, Ramphan S, Khongwichit S, Smith DR (2017). Activity of andrographolide against dengue virus. Antiviral Res..

[20] Shi TH, Huang YL, Chen CC, Pi WC, Hsu YL, Lo LC (2020). Andrographolide and its fluorescent derivative inhibit the main proteases of 2019-nCoV and SARS-CoV through covalent linkage. Biochem. Biophys. Res. Commun..

[21] Theerawatanasirikul S, Lueangaramkul V, Thangthamniyom N, Chankeeree P, Semkum P, Lekcharoensuk P (2022). Andrographolide and deoxyandrographolide inhibit protease and IFN-antagonist activities of foot-and-mouth disease virus 3Cpro. Animals.

[22] Wintachai P, Kaur P, Lee RCH, Ramphan S, Kuadkitkan A, Wikan N (2015). Activity of andrographolide against chikungunya virus infection. Sci. Rep..

[23] Ali S, Ahmad Mir S (2020). Antibacterial activity of *Andrographis paniculata* of methanolic extract against some human pathogenic bacteria and effect of andrographolide compound against bacterial pathogen. Int. J. Pharm. Sci. Res..

[24] Arifullah M, Namsa ND, Mandal M, Chiruvella KK, Vikrama P, Gopal GR (2013). Evaluation of anti-bacterial and antioxidant potential of andrographolide and echiodinin isolated from callus culture of *Andrographis paniculata* Nees. Asian Pac. J. Trop. Biomed..

[25] Ativui S, Danquah CA, Ofori M, Gibbons S, Bhakta S, Doe P (2022). Antibacterial and antifungal activities of andrographolide in combination with antimicrobial drugs. Res. J. Pharmacogn..

[26] Bassey K, Mamabolo P, Cosa S (2021). An Andrographolide from *Helichrysum caespitium* (DC.) Sond. Ex Harv., (*Asteraceae*) and its antimicrobial, antiquorum sensing, and antibiofilm potentials. Biology.

[27] Nidiry ESJ, Ganeshan G, Lokesha AN (2015). Antifungal activity of the extract of *Andrographis paniculata* and andrographolide. J. Pharmacogn. Phytochem..

[28] Yadav S, Ahmad F, Rathaur S (2022). Antifilarial efficacy of andrographolide: ex vivo studies on bovine filarial parasite *Setaria cervi*. Comp. Biochem. Physiol. C Toxicol. Pharmacol..

[29] Zaid OI, Abd Majid R, Sabariah MN, Hasidah MS, Al-Zihiry K, Yam MF (2015). Andrographolide effect on both *Plasmodium falciparum* infected and non-infected RBCs membranes. Asian Pac. J. Trop. Med..

[30] Page MJ, Moher D, Bossuyt PM, Boutron I, Hoffmann TC, Mulrow CD (2021). The PRISMA 2020 statement: an updated guideline for reporting systematic reviews. BMJ..

[31] Sheth VH, Shah NP, Jain R, Bhanushali N, Bhatnagar V (2024). Development and validation of a risk-of-bias tool for assessing in vitro studies conducted in dentistry: the QUIN. J. Prosthet. Dent..

[32] Sornambikai S, Amir H, Bhuvaneshwari G, Ponpandian N, Viswanathan C (2022). Review-systematic review on electrochemical biosensing of breast cancer miRNAs to develop alternative DCIS diagnostic tool. ECS Sens. Plus..

[33] Gopalakrishnan A, Maji C, Dahiya RK, Suthar A, Kumar R, Gupta AK (2016). In vitro growth inhibitory efficacy of some target specific novel drug molecules against *Theileria equi*. Vet. Parasitol..

